# From Mice to Mainframes: Experimental Models for Investigation of the Intracardiac Nervous System

**DOI:** 10.3390/jcdd8110149

**Published:** 2021-11-04

**Authors:** Matthew R. Stoyek, Luis Hortells, T. Alexander Quinn

**Affiliations:** 1 Department of Physiology and Biophysics, Dalhousie University, Halifax, NS 15000, Canada; Alex.Quinn@Dal.Ca; 2 Institute for Experimental Cardiovascular Medicine, University Heart Centre Freiburg–Bad Krozingen, 79110 Freiburg, Germany; luis.hortells@uniklinik-freiburg.de; 3 Faculty of Medicine, University of Freiburg, 79110 Freiburg, Germany; 4 School of Biomedical Engineering, Dalhousie University, Halifax, NS 15000, Canada

**Keywords:** neurocardiology, sympathetic, parasympathetic, mammal, zebrafish, cell culture, computational modelling

## Abstract

The intracardiac nervous system (IcNS), sometimes referred to as the “little brain” of the heart, is involved in modulating many aspects of cardiac physiology. In recent years our fundamental understanding of autonomic control of the heart has drastically improved, and the IcNS is increasingly being viewed as a therapeutic target in cardiovascular disease. However, investigations of the physiology and specific roles of intracardiac neurons within the neural circuitry mediating cardiac control has been hampered by an incomplete knowledge of the anatomical organisation of the IcNS. A more thorough understanding of the IcNS is hoped to promote the development of new, highly targeted therapies to modulate IcNS activity in cardiovascular disease. In this paper, we first provide an overview of IcNS anatomy and function derived from experiments in mammals. We then provide descriptions of alternate experimental models for investigation of the IcNS, focusing on a non-mammalian model (zebrafish), neuron-cardiomyocyte co-cultures, and computational models to demonstrate how the similarity of the relevant processes in each model can help to further our understanding of the IcNS in health and disease.

## 1. Introduction

The presence of an intracardiac nervous system (IcNS) has intrigued scientists for more than 200 years, with the first published reference by Scarpa in the *Tabulae Nevrologicae* in 1794 [1]. In the early 20th century, Cannon [2] and Langley [3] provided more detailed descriptions of the structural, functional, and pharmacological properties of visceral organ control by the autonomic nervous system (ANS). Traditionally, the ANS is described as an efferent system that provides control over visceral organ function that is critical for maintaining homeostasis, including that of the cardiovascular system. The ANS is composed of two principal efferent divisions, the parasympathetic and sympathetic nervous systems. Classically, regarding their organ function control, these divisions have been viewed as having a reciprocal antagonistic relationship [2,3]. The IcNS is the final common pathway for the ANS in the heart [4,5,6,7], and facilitates rapid, reflex-driven beat-to-beat modulation of cardiac output to match changes in systemic demand. Sometimes referred to as the “little brain of the heart” [8], the ICNS accomplishes this by modulating various aspects of cardiac performance, including chronotropy, dromotropy, lusitropy, and inotropy [8,9,10].

In the traditional model of cardiac regulation, the central nervous system (CNS), encompassing the spinal cord, medulla, and higher brain centres, processes cardiovascular feedback, and relays signals to the heart via peripheral ANS nerves. In this configuration, the IcNS is simply a conduit for central inputs to the heart [8,11]. The view of autonomic control of the heart has since evolved, and is now considered as organised hierarchically into three functional ‘levels’ [8]. Level 1 is composed of neurons within the CNS, typically the medulla and spinal cord, which are controlled by higher brain centres [8,9]. Level 2 is composed of intrathoracic ganglia (e.g., stellate ganglia), sensory nerves, sympathetic and parasympathetic efferent neurons, and connects the CNS to the heart. Level 3 is composed of those neural elements which are intrinsic to the heart itself (i.e., the IcNS) [8]. Recent advances have shown that the IcNS is far more complex than the simple ‘neuronal-relay system’ of classical dogma. Work in the last two decades has revealed neurochemical complexity [12,13], neuronal distribution [8,12,14,15], capability for local information processing, and neural interactions not explained by our traditional understanding of the IcNS [8,16]. In addition to the neuronal components, the CNS and ANS (including the IcNS) also contain myelinating and Remak Schwann cells (SC), neural-associated fibroblasts and perycites, macrophages, and endothelial cells [17,18]. It is assumed that the cells of the non-neuronal compartment of the ANS support neuronal development, impulse conduction, metabolic maintenance, and regeneration, but the available information regarding these cell types is surprisingly limited.

There exist numerous established experimental models that can be used to study the IcNS. In choosing an appropriate model, the similarity of the relevant processes to those in humans determines their utility for expanding our mechanistic understanding of human physiology and pathophysiology. In this paper, we review our current understanding of the anatomy of the principal components of extrinsic and intrinsic cardiac innervation. We then provide a brief overview of the knowledge derived from mammalian models (e.g., porcine, canine, and murine) and considerations for their use. We follow this with a description of alternative experimental models, focusing on zebrafish, cell culture, and computational models, to highlight how investigations using these models can further our understanding of IcNS structure and function.

### 1.1. Extrinsic Cardiac Regulation—The CNS and Intrathoracic Nervous System

The primary extrinsic control of cardiac activity is through the CNS, acting via extracardiac sympathetic and parasympathetic innervation of intracardiac neural circuits and cardiac tissues [19,20,21]. Preganglionic sympathetic neurons, typically originating from the reticular formation in the brainstem, including the ventrolateral medulla, project axons via the intermediolateral column of the spinal cord [8,22], exiting the spinal cord bilaterally and projecting to postganglionic intrathoracic neurons in the superior and middle cervical, cervicothoracic (stellate), and mediastinal ganglia [22,23,24], which project to the heart. Preganglionic parasympathetic neurons project axons from brainstem nuclei (e.g., dorsal vagal motor nuclei, insular cortex, nucleus ambiguus [8,9]) and travel via bilateral vagus nerves, and multiple cardiopulmonary branches, which form synapses with postganglionic parasympathetic neurons within cardiac ganglia [9]. Both the central (brain and spine) and intrathoracic (paravertebral ganglia) levels also receive information from afferent nerves originating from the heart, lungs, and vasculature [11].

It is worth noting that cardiac function may also be affected by neurohumoral agents (including peptides) produced in endocrine glands and released into the circulation [19,20,21]. Circulating epinephrine and norepinephrine released by the adrenal medulla (as well as dopamine, when converted to epinephrine or norepinephrine), will cause an increase in heart rate by a similar mechanism to neurotransmitters released locally by sympathetic neurons; however, the details of this are beyond the scope of this review.

### 1.2. Intrinsic Cardiac Regulation—The IcNS

It is now being shown that within the IcNS, in addition to intracardiac neurons, there also exist other cell types which play crucial roles in IcNS structure and function, including SC, small intensely fluorescent cells (SIF), and endoneurial fibroblasts (EF). Furthermore, it has been shown that the IcNS has the capability to exert reflex control of cardiac function, even when isolated from central and intrathoracic levels of the extracardiac ganglia and CNS [11,25,26,27].

#### 1.2.1. Intracardiac Neurons

Intracardiac neurons derive primarily from neural crest cells (NCC) that migrate to the developing heart (~5th week in humans and ~E8.5–9.5 in mice [10,28,29]) giving rise to sympathetic, parasympathetic, and afferent sensory neurons [10,28]. Currently much of what is known of embryonic and fetal development of IcNS neurons and their subsequent postnatal maturation is derived from studies in mice. Sympathetic intracardiac neurons originate from trunk NCC that first migrate ventrally towards the dorsal aorta, and then rostrally and caudally, forming the paravertebral sympathetic chain [10]. NCC, which ultimately derive sympathetic components, have been shown to reach the dorsal aorta and outflow tract between E9.5-E10.5 [29]. By E13.5-E15.5, sympathetic axons begin to extend along coronary veins and penetrate the subepicardium, but it is not until E17.5 that these axons infiltrate the myocardium [30]. Recently, it has been reported that sympathetic maturation is largely completed by P7 [31]. Parasympathetic intracardiac neurons originate either from cardiac NCC, a subset of the vagal neural crest [10], where they migrate laterally to the somites before entering the heart, or from cells within the nodose placode (~E12.5 in mice [10,28]). NCC, which will contribute to parasympathetic components, have been observed at the venous pole of the heart at E12.5 [28]; however, development of parasympathetic innervation is believed to occur postnatally from P0.5-P21 [32]. Maturation of parasympathetic innervation is still ongoing at P30 and the exact time course to reach a terminal maturation is currently unknown [31]. Development of cardiac neurons can be considered a co-maturation system, in which signals from cardiac tissue regulate the growth, patterning, and transmission properties of intracardiac neurons, while reciprocal signalling from intracardiac neurons influences the maturation of cardiomyocytes (CM) [33]. 

Intracardiac neurons can be broadly characterised by: (i) their anatomical/topographical layout [34]; (ii) their neurochemical phenotype [8,35,36]; and (iii) their functional influence on the heart [35]. Analogous to the enteric nervous system of the digestive tract, intracardiac neurons are typically found within an aggregation known as a “ganglionated plexus” (GP) [9,14,34]. The exact locations of GP, as well as their patterns of innervation to the atria and ventricles, are highly species-dependent, though generally GP are supraventricular and found on the epicardium in intramural tissue or in epicardial fat pads [9,10,16]. In terms of neurochemical phenotypes, it is now known that within the IcNS there are parasympathetic, sympathetic, and non-noradrenergic, non-cholinergic transmitters and modulators, and along with extrinsic inputs, intracardiac circuits may be important for internal signal processing and intracardiac reflex control [8,36]. Functionally, there are two broad classes of intracardiac neurons. Type 1, also referred to as phasic or somatic neurons, are typically monopolar (with a single axonal projection) and fire only a single action potential (AP) during a sustained depolarising stimulus. Type 2 intracardiac neurons are multipolar and fire multiple AP during a sustained depolarising stimulus [10,37]. Type 2 neurons can be further sub-classified as slow after-hyperpolarisation neurons or pacemaker neurons, which are distinguished by slow or rapid repolarisation characteristics, respectively [10]. A third type of intracardiac neuron has also been demonstrated, referred to as an accommodating neuron, which fires multiple APs at a decreasing rate during sustained depolarisation [37].

Functional data have shown that intracardiac neurons receive inputs from extrinsic efferent parasympathetic preganglionic and sympathetic postganglionic neurons, local circuit neurons, and afferent neurons from locations across the heart [37,38]. As a result, even after acute [38] and chronic [25] isolation of the heart from the CNS (“decentralisation”), the IcNS remains responsive to changes in the cardiac environment. In addition, by simultaneous recording it has been demonstrated that parasympathetic (vagal) and sympathetic (cardiopulmonary) nerves can be co-activated [39,40,41], and that adrenergic-cholinergic interactions can augment activity of the IcNS in a manner that does not occur in the presence of only one input [37]. This may be less surprising when one considers that it has been shown that there are intracardiac neurons which respond weakly, or do not respond, to direct vagal stimulation simulating CNS input [8,25].

#### 1.2.2. Intracardiac Schwann Cells (SC)

SC in the heart are neural crest derived glial cells that are necessary for nerve fasciculation in development [31]. While whole heart expression patterns are not currently known, electron [17] and fluorescent [31] imaging have described the presence of two distinct SC populations: (i) a small population of myelinating SC [42] and (ii) a prevalent population of non-myelinating cells, known as Remak SC, which are thought to play a role in development and regeneration [31,43]. An initial description of cardiac SC gene expression in homeostasis has been shown by single cell RNA sequencing [44], but the role of these cells in cardiac disease remains unexplored. 

#### 1.2.3. Small Intensely Fluorescent (SIF) Cells

SIF cells have long been known to exist within the CNS, and while they have been described in the ganglia of the heart [12], their presence remains incompletely understood. It is known that these small ‘neuron-like’ cells are catecholaminergic, with strong immunoreactivity for tyrosine hydroxylase (TH; the rate limiting enzyme in catecholamine synthesis [12,45]). While the presence of TH confirms a catecholaminergic phenotype, these cells lack immunoreactivity to dopamine beta-hydroxylase, the enzyme involved in the conversion of dopamine to norepinephrine. In some cases, they exhibit immunoreactivity to tryptophan hydroxylase, which is involved in the synthesis of serotonin (or 5-HT). Further to this, it has been reported that cholinergic synaptic terminals are frequently observed near SIF cells [12], and it has been proposed that SIF cells may have endocrine, chemoreceptive, or interneuronal roles [10]. Ultimately, the origins and role of SIF cells within the IcNS remains to be explored.

#### 1.2.4. Intracardiac Endoneurial Fibroblasts (EF)

While they have been known in other organs for decades, EF had not been described in the heart until recently [31]. Aggregations of EF around thick nerves of the atria, outflow tract, and epicardium were found to be of neural crest origin, while EF that supported thinner intraventricular nerves are probably derived from epi- or endocardial precursors [31]. It is assumed that EF play a crucial role in nerve extracellular matrix homeostasis, but due to the lack of experimental tools such as cell-specific markers, there is only minimal data available regarding this cell type in the heart.

### 1.3. Therapeutic Potential of IcNS Targets

In recent years our understanding of the specifics of ANS control of the heart has greatly expanded and the ANS is increasingly being implicated in cardiovascular disease [8,9,46,47]. However, investigation of the specific functional roles of subpopulations of intracardiac neurons has been hampered by a lack of knowledge regarding the functional network interactions of the neural and non-neural compartments of the IcNS. Multiple levels of the CNS and IcNS, comprising several integrated feedback loops, are involved in the regulation of ANS function and may be disturbed in pathology. These include intracardiac cardio-cardiac reflexes and intrinsic cardiac nerve activity that alter nerve transmission within the myocardium, intrathoracic reflexes and feedback mechanisms that modify sympathetic ganglionic efferent transmission, and spinal, lower brainstem, and higher brain centre regulation that modulates autonomic outflow [48,49,50]. 

Chronic alterations in sympathetic/parasympathetic balance are a well-established contributor to many cardiovascular diseases, being strongly linked to clinical outcome and prognosis [48,51], and emerging clinical data highlight a role for intracardiac neurons in ANS imbalance and the resulting disease progression [51].

It has also been shown that the IcNS may play a role in genetic disorders (e.g., Brugada and Long QT syndrome), neurodegenerative conditions (e.g., epilepsy, dysautonomia), myocardial injury, acquired arrhythmias (such as atrial fibrillation), and contractile dysfunction [52]. Furthermore, associated neural remodelling [53] can alter ion channel function and signalling cascades, and thus impact cardiac function [33,52]. Currently, most therapeutic interventions in pathological states such as heart failure and arrhythmia are pharmacologic and impact the entire neurohormonal axis [9]. More targeted neuromodulatory treatments are currently being explored in pre-clinical and clinical studies, including vagal nerve stimulation, cardiac sympathetic denervation, renal denervation, spinal cord stimulation, baroreflex activation, neurotoxin injection, and tragus stimulation [47,54]. 

In the setting of heart transplantation, where the donor heart has been decentralised, reinnervation may occur in an inconsistent and incomplete manner [55]. This decentralisation has been related to a depletion in circulating catecholamines, reduced exercise capacity, increased resting heart rate, and disturbed blood pressure regulation [55,56]. Thus, it is clear that a comprehensive understanding of how the nervous system controls the heart, and what can go wrong with this control system, is a key factor in developing successful new neuronally-based cardiac therapies [54,57].

## 2. Mammalian Models of the IcNS

Mammalian models, including large animals (e.g., pigs, sheep, dogs), smaller animals (e.g., rabbits), and most commonly rodents (e.g., mice, rats, and guinea pigs) have long been the mainstays for experimental neurocardiology.

### 2.1. Anatomy of the IcNS

**Figure 1:**
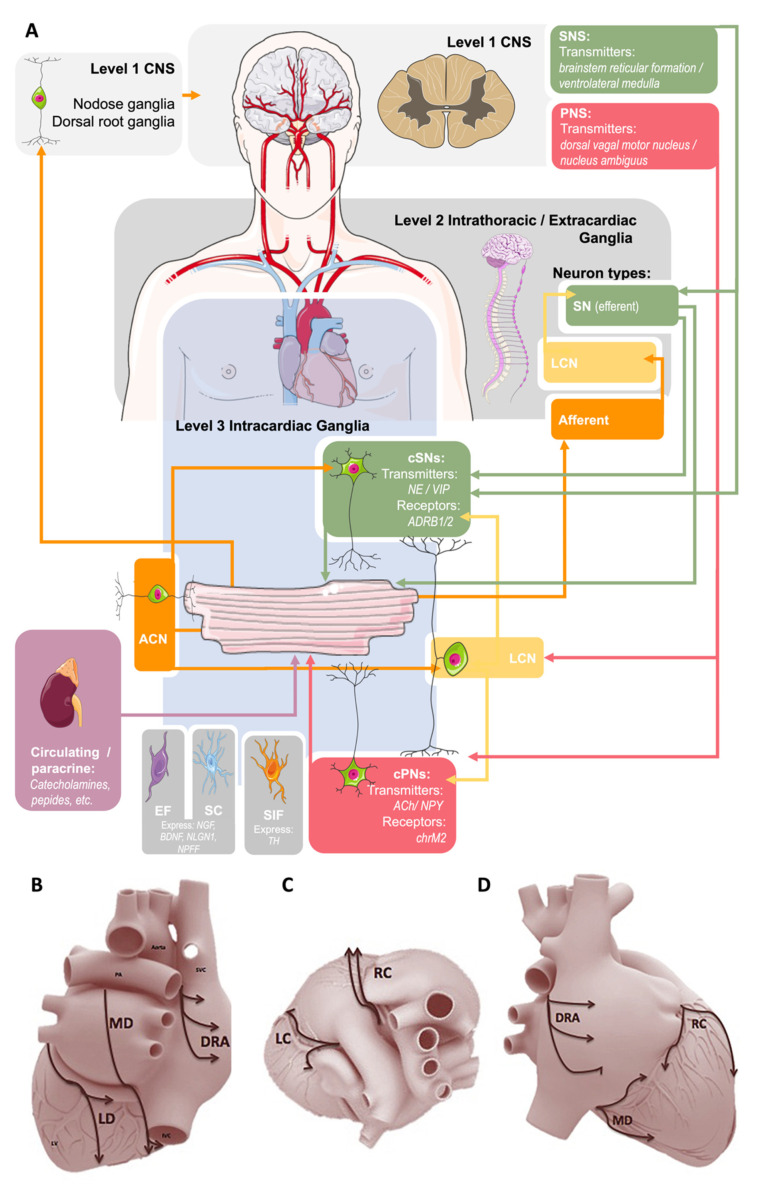
Summary of the current conceptual model of autonomic control of the heart. (**A**)**:** Sympathetic innervation (SN, green) originates from the central (Level 1) and intrathoracic (Level 2) levels and projects to cardiac sympathetic neurons (cSNs) within the intracardiac nervous system (IcNS, Level 3). Parasympathetic (PNS) innervation (red) comes from the central level via the vagus nerve to cardiac parasympathetic (cPNs) and local circuit (LCN) neurons within the IcNS. Sensory afferent neurons (orange) project to nodose and dorsal root ganglia, and afferent cardiac neurons (ACN) project to SCNs and LCNs. Arrowheads indicate direction of nerve conduction. At the level of the IcNS there are also small, intensely fluorescent (SIF) cells, Schwann cells (SC), and endoneurial fibroblasts (EF). Parts of the illustration were adapted from Servier SMART under a CC BY 3.0 license. (**B**)**:** A proposed scheme of innervation of the mammalian heart by the cardiac ganglionated plexuses in dorsal (**B**), right lateral (**C**), and superior (**D**) views. Ganglia: DRA, dorsal right atrial; LC, left coronary; LD, left dorsal; MD, middle dorsal; RC, right coronary. Reproduced with permission from [22].

**Figure 2:**
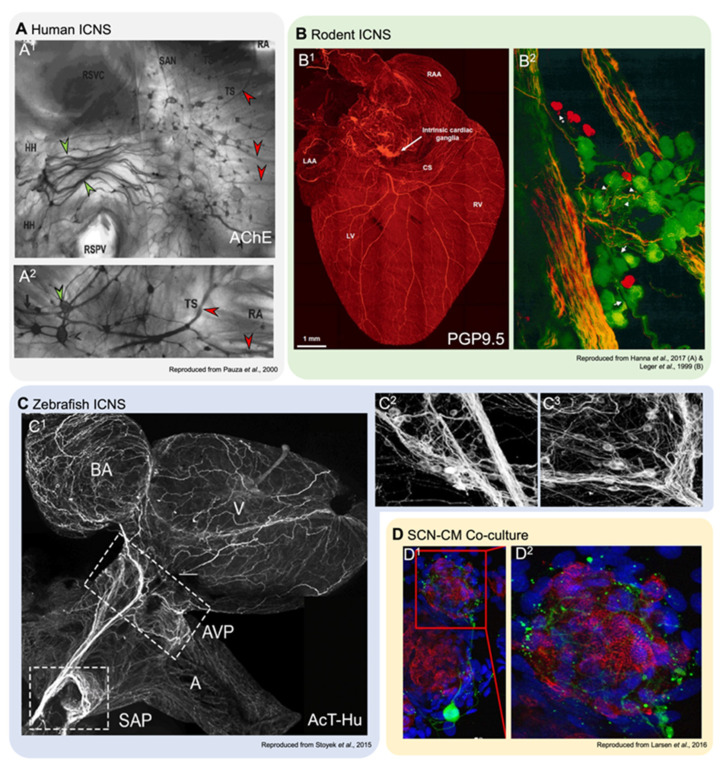
Innervation of cardiac tissues in human and in experimental models. (**A^1,2^**)**:** Acetylcholinesterase staining of right atrial human atrial innervation. Thicker nerves situated over the ganglia (green arrowheads), which are stained for AChE, are paler than the smaller ganglia (red arrowheads; reproduced with permission from [15]). HH, heart hilum; RA, right atrium; RSPV, right superior pulmonary vein; RSVC, root of the superior vena cava; SAN, sinoatrial node; TS, terminal sulcus. (**B^1,2^**)**:** Dorsal view of a mouse heart (**B^1^**) stained with pan-neuronal marker PGP9.5 (protein gene product 9.5; reproduced with permission from [50]), and a detailed view of a left atrial ganglia from a guinea pig (**B^2^**) stained with PGP9.5 (green) and TH (red), showing neurons and SIF cells (reproduced with permission from [12]). CS, coronary sinus; LAA, left atrial appendage; LV, left ventricle; RAA, right atrial appendage; RV, right ventricle. (**C^1−3^**)**:** Overview of innervation in the zebrafish heart (**C^1^**) and detail-views of left (**C^2^**) and right (**C^3^**) vagal ganglia, stained with pan-neuronal acetylated tubulin and human neuronal protein C/D (reproduced with permission from [64]). A, atrium; AVP, atrioventricular plexus; BA, bulbous arteriosus; SAP, sinoatrial plexus; V, ventricle. (**D^1,2^**)**:** Co-cultures of rat ventricular myocytes and sympathetic stellate neurons. Alpha actinin marks myocytes (red), TH (green) is a sympathetic neuron marker, and nuclear marker DAPI (blue; reproduced with permission from [51]).

GP from small mammals appear to be discretely located (although their arrangement is species-dependent), but are more diffusely distributed in larger mammals. Based on neuronal inputs and intracardiac projections to effector cells, GP have been generalised into five to seven regions (Figure 1): (i) right dorsal atrial; (ii) ventral right atrial; (iii) left dorsal; (iv) ventral left atrial; (v) middle dorsal; (vi) right coronary; and (vii) left coronary [9]. GP in all of these regions have been demonstrated in the rabbit [58], dog [14], sheep [59], and human [15,18]. Intrinsic cardiac nerves extend epicardially from GP to innervate the atria, interatrial septum, and the ventricles [18,59,60] (Figure 2A,B). Two subplexus routes extend from the arterial region of the hilum between the pulmonary trunk and the aorta to effector sites on the left and right ventricles. A further five sub-GP are found in the venous region of the heart, including: (i) dorsal, at the right caudal vein or the superior vena cava; (ii) middle dorsal, at the pulmonary veins; (iii) left dorsal; (iv) ventral left atrial; and (v) right ventral. The defined routes of these GP are comparatively similar amongst mammals [9]. A complete mapping of the GP and their projections can be found in the review by Wake and Brack [35]. 

In contrast to our understanding of cardiac innervation of the atria, that of the ventricles remains less well understood and underappreciated. Mammalian ventricles were historically believed to be devoid of ganglia and innervation from the IcNS until ventricular ganglia were found in the dog [61] and human [38] at the coronary groove and around the conus arteriosus (CA). These findings have since been replicated in humans [18], sheep [59], dog [14], mice [62,63], and rabbit [58,60]. A more detailed investigation of the extremely rich intraventricular innervation has been performed recently using high-resolution fluorescence microscopy, which suggests there are direct neuronal effects on CM through neurotransmitter release from nearby varicosities [35].

In terms of neurotransmitters, it has been shown that sympathetic cardiac neurons generally contain norepinephrine, and in some instances the co-transmitters ATP, neuropeptide-Y (NPY), and galanin [33]. Parasympathetic cardiac neurons contain acetylcholine and co-transmitters such as natriuretic peptide Y, somatostatin, vasoactive intestinal polypetide, and endogenous opioids [33]. The presence of small catecholaminergic SIF cells associated with cholinergic innervation has also been described within the IcNS of guinea pig, which could modulate cardiac neural activity [12]. Moreover, a recent study described neuronal somata that exhibit co-localised immunoreactivity for choline acetyltransferase (ChAT, a rate limiting enzyme in the synthesis of acetylcholine) and TH within ganglia of the mouse heart [63], suggesting that parasympathetic and sympathetic transmitters may occur within the same intracardiac neurons [33].

### 2.2. Function of the IcNS

Acute [38] or chronic [25,27] decentralisation of the heart from the CNS has shown that intracardiac neurons in the mammalian heart remain viable and responsive to changes in the cardiac milieu. A complex and integrative view of the autonomic control of cardiac function has thus emerged, supplanting beliefs that individual GP solely innervate regions adjacent to the GP. It is now well-documented that GP can innervate and alter the function of both proximal and distant regions via intra- and inter-ganglionic communication [65,66,67]. The coordination of neuronal inputs and outputs within the IcNS depends on the nature of the afferent nerve supply, the efferent neuronal inputs received from the central/peripheral nervous systems through the sympathetic and parasympathetic nervous systems, and intrinsic connections via local circuit neurons within the IcNS.

An example of this is found in the posterior atrial GP, which receives inputs from sympathetic fibers from the right stellate ganglion, while parasympathetic intracardiac neurons from that GP project to the SAN and exert a negative chronotropic effect [68,69]. Thus, while it may be tempting to imagine that a specific IcNS GP (e.g., the right atrial GP) could be ablated to affect a particular cardiac region (e.g., the SAN), pleiotropic effects of GP result in off-target effects [9,70,71]. In fact, it has been shown that direct electrical stimulation of the cardiac vagal nerves (to simulate CNS input) results in weak evoked potentials in some intracardiac neurons in the beating mammalian heart, but these fail to fire action potentials (i.e., subthreshold stimulation) [8,37,72,73,74,75,76]. From those findings it is hypothesised that these ‘orphaned’ intracardiac neurons, which lack extrinsic input, may be acting as local circuit neurons. As more refined methodologies develop, researchers are now utilising molecular analyses to better characterise differences in intracardiac neuron populations. For instance, a recent study provided the first report of single cell RNA sequencing and functional analysis of stellate ganglia neurons, validated against human stellate ganglia neurons, in an effort to better characterise neurons innervating the heart [77].

### 2.3. Considerations for Investigations Using Mammalian Models

A major issue requiring further clarification for understanding the control of cardiac function by the IcNS is the distribution and connectivity of intracardiac neurons. The IcNS is estimated to comprise 43,000–94,000 neurons in the human heart, ~80,000 in dog, ~12,000 in pig, ~2200 in rabbit and guinea pig, ~6500 in rat, and ~1000 in mouse [35]. In the mammalian heart, intracardiac neuronal somata are widely distributed in ganglia throughout both atria [12,13,14,17,58,63], but with few exceptions (i.e., dog [78] and guinea pig [13]) the projection patterns of subpopulations of intracardiac neurons to specific targets remain largely unknown.

In mammalian hearts, a major difficulty in studying the role of specific components of the IcNS is their visualisation (Figure 2A,B) and manipulation. The IcNS is deeply embedded in, and distributed throughout, the walls of both the atria and ventricles, and thus largely inaccessible for integrative studies. One solution to this problem is the use of ‘reduced’ experimental preparations representing a subset of the IcNS, such as isolated tissues containing intracardiac neurons in GP, to study properties of control circuits in vitro [25,27]. This approach, however, eliminates a majority of intracardiac neuronal connections, so only limited conclusions may be drawn about the roles of localised circuitry in controlling overall cardiac function.

For structural microscopy studies, recently developed techniques such as optical clearing protocols, have been successfully applied to hearts of smaller mammalian models [79,80,81]. Even with small hearts, this method can take weeks to process, requires highly specialised imaging equipment, and involves time-consuming and technically challenging sequential imaging and reconstruction approaches to provide a view of cardiac innervation on an organ level. Most importantly, as it is conducted on fixed tissue, clearing-based imaging yields structural information only.

## 3. Zebrafish for Investigations of the IcNS

One of the first experimental models used to investigate cardiac innervation was the frog [82]. The frog IcNS contains approximately 1300 neurons, which express both cholinergic and adrenergic transmitters. Neurons are distributed in ganglia in the interatrial septum, atrioventricular junction, and sinus venosus [82]. However, due to limitations of the model, including a relatively simplistic neuronal network (each intracardiac neuron receives innervation from only one to two preganglionic axons [82,83]), and the move toward more genetically conserved models, the use of the frog is no longer common [82].

Over 30 years ago the zebrafish began to gain attention as a model for experimental research, primarily owing to its conserved genetics [84,85,86]. The high fecundity, relatively fast generation time, and external embryogenesis of the zebrafish, along with nearly optically transparent embryos that permit detailed in vivo observations down to the subcellular level during development [84,87], has led to a growing adaptation of the zebrafish as an experimental model. For cardiac-specific investigations, the zebrafish is becoming an increasingly powerful model owing to: (i) its similarities to humans in terms of both genetic and functional properties (comparable heart rate, AP morphology, and ion channel and Ca^2+^-handling protein expression and function); (ii) a fully sequenced genome that can be easily manipulated by standard techniques [88,89]; and (iii) the ability of researchers to visualise the IcNS and cardiac function in vivo during development and in situ in the isolated adult heart [26,64,87,88,90,91,92,93,94] (Figure 2C). Numerous studies in zebrafish have generated considerable advances in understanding mechanisms underlying (patho-)physiological cardiac function, but few have considered the role of integrated ANS control of the zebrafish heart. 

### 3.1. Zebrafish IcNS Anatomy

Despite the advantages of the zebrafish for developmental studies, there is a paucity of reports describing IcNS anatomy and the mechanisms of its development in embryonic zebrafish [95]. While the presence of parasympathetic (e.g., M_2_ muscarinic receptors) and sympathetic (e.g., β-adrenergic receptors) receptors in the larval zebrafish have been established [96,97], and their functional relevance has been substantiated by pharmacological studies [98], specific details of the structure and function of this system in development remain to be described. 

In contrast, the adult zebrafish has been used to overcome limitations of larger animal models for studies of both extrinsic and intrinsic cardiac regulation involving the IcNS [26,64]. Within the zebrafish IcNS there are six to eight loosely formed cardiac GP: the proximal (i) left and (ii) right vagal ganglia within the venous sinus-atrial wall; (iii) the dorsal ganglion within the SAN region; the (iv) left and (v) right vagal ganglia at the junctions of the venous sinus and SAN region (where the vagal nerves enter the heart); the (vi) left and (vii) right ventral ganglia within the SAN region; and (viii) 1–2 atrioventricular ganglia at the atrioventricular valves [64]. Overall, it has been shown that approximately 90% of all intracardiac neurons present in the zebrafish heart are located within cardiac ganglia adjacent to the SAN.

All GP surrounding the SAN of the zebrafish are innervated by extrinsic axons from the left and right vagal nerves (termed vagosympathetic nerves, as both sympathetic and parasympathetic travel together in this species; Figure 2C), with synaptic terminals concentrated near intracardiac neurons or adjacent to putative pacemaker cells [26,64]. As would be predicted by the traditional view of the IcNS, it has been shown that the vast majority of intracardiac neurons within zebrafish GP are cholinergic, however some are putative adrenergic TH-positive neurons. There are also lateral differences in projection patterns of vagal axons to the GP; axons from the right trunk target discrete regions within the SAN, while axons from the left vagal nerve extend around the SAN and form a nerve trunk projecting to the atrioventricular region [64].

In addition to cholinergic and adrenergic neurotransmitters, other ‘non-canonical’ (non-adrenergic, non-cholinergic) autonomic transmitters have been detected within the zebrafish IcNS. Neuronal nitric oxide synthase is present in a small subset of SAN intracardiac neurons, serotonin can be found in intracardiac neurons, ‘glial-like cells’, and cells associated with the endocardium of the atrium, and vasoactive intestinal polypetide is present in nerve terminals [64,99]. The role of neurons secreting these transmitters in the zebrafish heart is not clear, but it has been suggested that intracardiac release of nitric oxide is involved in modulating cardiac effector cells, while serotonin may have a role in neuronal regulation and development [99].

A particularly intriguing finding from studies in zebrafish is the detection of what appears to be neuronal cell bodies in the IcNS, with axons projecting back to the central nervous system. These intracardiac neurons may represent afferent neurons, similar to anatomical evidence shown previously in the rat heart [100]. If these neurons in the zebrafish heart are in fact afferent neurons, they may provide cardio-sensory input from local, intracardiac control circuits to centrally mediated control mechanisms [26].

### 3.2. Zebrafish IcNS Function

Following these anatomical studies in zebrafish, functional investigations in isolated hearts with intact extrinsic innervation established the zebrafish as a novel model for studies of autonomic control of SAN function [26]. Using electrocardiogram (ECG) and voltage optical mapping recordings during extrinsic vagal nerve stimulation and pharmacological interventions, it was shown that discrete neural pathways modulate SAN function through adrenergic and cholinergic mechanisms in a similar manner to mammals (effects which are present as early as 4·days post-fertilisation [98]). Expression of adrenergic and cholinergic receptors in SAN cells was shown via immunohistochemistry, supporting the pharmacological data, and suggesting SAN cells are under the direct influence of the IcNS [26]. Further, responses to individual or simultaneous right and/or left vagal nerve stimulation revealed a summative decrease in heart rate, indicating that each vagal nerve activated different groups of post-ganglionic ‘rate-control’ neurons within the IcNS, in agreement with evidence from the mammalian heart [26]. Vagal nerve stimulation at an intensity that evoked significant bradycardia also elicited a variable post-stimulus tachycardia, a ‘biphasic’ response that is also present in mammals [101]. Finally, strong simultaneous vagal nerve stimulation in the zebrafish altered the pattern of initiation and propagation of SAN excitation, again similar to what is observed in mammalian hearts [26,102,103].

It has been shown that the IcNS in zebrafish is not a static system. It appears that the IcNS matures relatively slowly in zebrafish, as the total number of intracardiac neurons increases until 12 months post fertilisation (a time-point well past sexual maturation, which occurs ~3 months post fertilisation [104]). However, at the same time, vagal nerve stimulations show a decrease in cardiac response with age, a situation similar to that observed in humans [104].

Combined, the above studies represent a first step towards establishing the use of the zebrafish as an experimental model for the study of the integrated control of cardiac function by the IcNS. This model may now be utilised to identify specific relationships between subpopulations of intracardiac neurons and their targeted effector cells—which have been elusive in other experimental models—to discover basic mechanisms of intracardiac neural regulation.

### 3.3. Considerations for IcNS Investigations in Zebrafish

While similarities in cardiac function and its regulation exist between mammals and zebrafish, there are morphological and systemic differences between zebrafish and mammalian hearts (e.g., 2 vs. 4 chambers, ring- vs. sheet-like SAN structure, low vs. high blood pressure [105]) which should be considered when using this model. In terms of IcNS function, much of what is known about the basic function and mechanisms from studies of mammalian intracardiac neurons has yet to be studied in the zebrafish. Currently, there have been no electrophysiological studies of the basic function and biophysical properties of the neurons within the zebrafish IcNS at any stage of development. 

The overall size of the zebrafish heart, ranging from the µm scale in juveniles (<3 months post fertilisation) to mm scale in the adult (≥3 months post fertilisation), presents technical challenges for standard electrophysiology (e.g., ECG leads [20,26,104]) and limited tissue availability for molecular analyses. Conversely, the small size and resulting accessibility for experimental manipulation becomes an advantage when performing immunohistochemical investigations, functional fluorescence imaging [104,106], or optogenetic studies [107], as it permits the visualisation of the IcNS, from individual intracardiac neurons down to the level of synapses and receptors in the whole heart, without being hindered by light penetration into cardiac tissue, as in mammals [26,64,104]. 

To understand the potential utility of the zebrafish for genetic studies, one must consider that owing to a whole genome duplication, of the 71% of conserved genes between human and zebrafish, 24% have multiple orthologs, which can confer redundancy in gene function and confound certain manipulations [88], potentially limiting direct translation of findings to human. Overall, though, the degree of genetic conservation has permitted the zebrafish to be a useful model in genetic manipulation studies and there have been many successful applications of genetically modified zebrafish in the fields of neuroscience and cardiovascular research [88,108]. An exciting example of this is the emerging trend to ‘humanise’ the zebrafish by driving them to express human genes (typically by knock-in strategies or editing of zebrafish orthologs to resemble more closely human genes) [88,108]. While studies using stem cells have proven a useful tool for this, zebrafish are increasingly being employed, owing to the ability to study genes of interest in a complex in vivo and native cardiac environment.

In the zebrafish, cardiac vagosympathetic nerve stimulation will activate both parasympathetic or sympathetic components of the IcNS, and specific activation of either has thus far only been accomplished pharmacologically [26,98,104]. With rapid advances in genetic manipulation, it is plausible that experimental tools such as optogenetics, to which zebrafish are a highly amenable model [107], will permit specific parasympathetic and sympathetic control of the IcNS. The zebrafish IcNS has also been shown to respond robustly to several known clinical pharmacological agents, ranging from serotonin reuptake inhibitors [99] to inhalational anesthetics [109]. It has been shown however, that vagal nerve stimulation appears not to affect some key cardiac indices in the zebrafish, such as atrioventricular delay and ventricular contractility, even though it is known to do so in mammalian models [26,104], suggesting an additional limitation to the use of zebrafish for such studies. 

Limitations aside, overall, it has been established that the zebrafish appears to retain all critical components of extrinsic and intrinsic IcNS regulation observed in mammals, making it a powerful emerging experimental model for studies of integrated IcNS physiology and pathophysiology.

## 4. Cell Culture and Computational Modelling Studies

While the use of model organisms for in situ studies of the IcNS has been the mainstay, the move to in vitro studies using neurons co-cultured with CM (frequently generated from induced pluripotent stem cells, iPSC), and in silico studies using computational models, are important recent advances.

### 4.1. Neuron-CM Co-Cultures to Study the IcNS

Co-cultures of sympathetic neurons and CM have been investigated for over 30 years [110,111], however past studies were frequently carried out at a microscopic (single cell) scale, where effects on cardiac function cannot be assessed directly [112]. CM monocultures imaged at macroscopic scales have allowed for the investigation of more complex functional tissue-level properties [113] and arrhythmia mechanisms [112]. Functional co-cultures of sympathetic neurons and CM (Figure 2D) offer the opportunity to study: (i) sympathetic neurons and CM function in a more complex environment; (ii) the influence of sympathetic neurons on CM functional properties; and (iii) the effects of neuronal density on co-cultured CM function [112].

Pluripotent stem cells have emerged as an important source of human cells in which cellular development and function can be studied in vitro under physiological and pathophysiological conditions [114]. Using human-iPSC (hiPSC), hiPSC-derived sympathetic neurons have been characterised in monoculture and in co-culture with hiPSC-derived CM [114]. It has been shown that the differentiation of hiPSC into norepinephrine-secreting cells produces sympathetic neurons that are able to modulate the beating rate of co-cultured CM [114]. It was found that nicotine exposure significantly increased CM beating rate in sympathetic neuron-CM co-cultures, but not in CM monocultures, suggesting a direct influence of nicotinic sympathetic neuron stimulation [114]. Fluorescence labelling revealed post-synaptic nicotinic acetylcholine receptors co-localised to TH-positive somas and synaptic varicosities in sympathetic neurons, which mediate the effect of nicotine. [114]. It has been shown that interactions with CM can alter the function of sympathetic neurons, including: (i) improved AP kinetics of mature hiPSC-derived sympathetic neurons compared to age-matched neurons in monoculture; and (ii) the promotion of neuronal maturation and increased expression of norepinephrine, nicotinic receptors, and relevant ion channels [114].

In terms of studying pathophysiology, recent reports have shown that intracardiac neuronal activation in co-culture can provide protection from arrhythmogenic behavior of CM [112,114]. In an elegant display of the versatility of such co-cultures, diseased stellate neurons and CM from healthy rats or from rats predisposed to hypertension were co-cultured, which demonstrated that cross-culturing pre-hypertensive diseased stellate neurons provokes CM pathology, while co-culturing healthy neurons with CM from pre-hypertensive rats rescues CM function [48,51].

### 4.2. In Silico Investigations of IcNS Function

Based on the multi-level (central, thoracic, and intracardiac) model of the ANS [8], a computational model has been designed to evaluate dynamic neural network activity and communication between the hierarchy of cardiac neuronal centres [11,115,116,117]. In the model, neurons at every level act both individually and in concert with other neurons at the same level [11], under the assumption that neurons within the network: (i) do not respond en masse as a single unit; and (ii) can influence others or be influenced by others through connections between and within levels (“plasticity”). Findings of these studies suggest that intracardiac neuronal activity becomes deranged with pathological insults, and that in pathophysiological conditions (such as ischemia [115]), neurons at all levels become dysfunctional. As a result, communication between the three levels of ANS control becomes deranged and the overall neural control of the heart is reduced [115]. It has also been shown that changes in the demand for cardiac output may broadly alter neuronal network properties in the IcNS beyond the activation of individual intracardiac neurons. The effect of constitutive alterations of individual neurons’ function introduces a degree of plasticity to the system, though ultimately it is a net ‘consensus signal’ that is transmitted to the heart to affect a change in its beating rate [116]. From this model it appears that the neural network may act as a protective ’buffer’ by filtering excessive central sympathetic drive during pathological changes. Furthermore, it was shown that dynamic neuronal networking within the IcNS could act to extinguish low-frequency oscillations in heart rate, a feature of particular interest in clinical settings, where such oscillations are taken as an indicator of cardiovascular disease [116,117].

### 4.3. Considerations for Co-Culture and Computational IcNS Models

To date, the electrophysiological properties of hiPSC-derived sympathetic neurons in neuron-CM co-cultures remain largely uncharacterised. Current techniques for generating co-cultures rely on the harvesting of CM from neonatal animals, and it should be recognised that such neonatal cells exhibit a different morphology and phenotype compared to mature cells [112]. At the same time, the correct proportion of neuronal cells/fibers, different types of innervation (e.g., parasympathetic, sympathetic, sensory), CM, and other cell types (e.g., fibroblasts, endothelial cells, SC, EF) is essential for accurately recapitulating the native tissue setting, and an incorrect balance may lead to aberrant cellular function and faulty conclusions [55]. Although co-cultures have clear advantages over the use of single-cells and monocultures, correlation of observed events with in vivo activity will require further validation (e.g., simultaneous paired sympathetic neuron-CM recordings [114]), to better understand potential differences between the in vitro cells and native cardiac tissue [112].

While co-cultures of sympathetic neurons and CM are well established, it appears that currently there are no published protocols for the differentiation of iPSC to parasympathetic neurons [55], thus representing an area for further research. There have been conflicting reports of the ability of hiPSC to be differentiated into sympathetic neurons capable of modulating co-cultured CM. It has been observed that hiPSC can be differentiated into norepinephrine-secreting sympathetic neurons capable of modulating the contraction rate of co-cultured neonatal mouse ventricular CM, but they lack functional coupling between sympathetic neurons and CM, while others have demonstrated sympathetic-like neurons without measurable norepinephrine secretion but with moderate effects on CM beating rate, putting the functional maturation of stem cell-derived cells, including CM, into question [114].

In terms of computational models, it has been acknowledged that in the model described above [11], heart rate changes and the accompanying changes in blood supply to the heart would subsequently alter cardiac output and performance, presenting a tremendous theoretical challenge for the model [116]. At the same time, the model was designed to interrogate neuronal interactions and their (dys-)function in specific pathophysiological conditions, so it may not be appropriate for fundamental studies of the IcNS.

Neural networks involve not only the synaptic relationships within a population of neurons, but also the intrinsic properties of each neuron within that population [11]. The focus of computational models thus far has been phenomenological in nature, investigating the effects of interactions among neurons between and within the cardiac centres, and not on the function of the neurons themselves. IcNS computational models have not advanced to the same extent as other cardiac computational models, in which it is possible to alter specific biophysically-based parameters to understand particular features of health and disease [118,119]. At the same time, the resolution of available computational IcNS models is also not at the level of other cardiac computational models [116], which, while acceptable for phenomenological networking studies, will require refinement for modelling specific, individual cardiac neuron parameters.

## 5. Conclusions

The IcNS regulates cardiac function via reflex pathways between the CNS and the heart, as well as within the heart itself [9]. While mammalian studies of the IcNS have long been mainstays of the field, the size of the heart and nature of the IcNS in these models has resulted in studies utilising reduced preparations, with truncated network connections, which has limited integrative insight. Zebrafish offer many advantages, including observation of in vivo and developmental aspects not possible in mammalian models, however the basic anatomy and physiology of the zebrafish means that caution should be exercised in extrapolating results to mammals. Co-culture systems and computational modelling offer novel possibilities for studies of the IcNS, however at present both have been utilised to investigate only minimal cell types and cellular interactions.

Understanding the diversity of neuronal and non-neuronal cell types within the IcNS, their interactions, and how they are involved in cardiac control is vital to our understanding of IcNS function and its role in cardiac pathologies. A more complete picture of the neurotransmitter/neuromodulator profiles of neurons within the IcNS, the anatomical and functional connectivity between GP within the heart and between GP and the ANS, and the extent to which these components can affect cardiac activity is needed [35]. Ultimately, the potential for crosstalk within the neural and non-neural compartments of the IcNS, the presence of mixed neuronal and non-neuronal populations within the GP of the IcNS, and the distributed nature of the network, may present multiple targets for neuromodulatory interventions to treat cardiovascular pathologies [9]. Thus far, there is no one satisfactory model to tackle the huge challenge of quantitatively exploring the mechanisms and effects of the IcNS on cardiac structure and function, but a diverse array of IcNS experimental models is now available. Perhaps combined, they will lead to a more comprehensive understanding of the IcNS, from the function of individual neurons to its network activity.
